# Association of Adverse Childhood Experiences and Metabolic Syndrome: A Systematic Review and Meta‐Analysis

**DOI:** 10.1111/obr.70095

**Published:** 2026-01-27

**Authors:** Joohan Kim, Luyu Xie, Alejandra Fernandez, Jaime P. Almandoz, Sarah E. Messiah

**Affiliations:** ^1^ Texas A&M University College Station Texas USA; ^2^ Center for Pediatric Population Health University of Texas Health Science Center at Houston (UTHealth) School of Public Health Dallas Texas USA; ^3^ Department of Epidemiology, Human Genetics and Environmental Sciences UTHealth School of Public Health Dallas Texas USA; ^4^ Department of Health Promotion and Behavioral Sciences UTHealth School of Public Health Dallas Texas USA; ^5^ Department of Internal Medicine, Division of Endocrinology University of Texas Southwestern Medical Center Dallas Texas USA; ^6^ Department of Pediatrics McGovern Medical School Houston Texas USA

**Keywords:** adverse childhood experiences, childhood maltreatment, childhood trauma, metabolic syndrome

## Abstract

**Background:**

The association between adverse childhood experiences (ACEs) and metabolic syndrome (MetS) across life course and its components (abdominal obesity, elevated blood pressure, dyslipidemia, and hyperglycemia) is poorly understood.

**Methods:**

Three databases were screened for studies published January 2000–February 2024 that examined the association between ACE and MetS. Relevant data, including authors, country, study type, participants, types, and number of ACE and MetS and its components, were extracted. Mantel–Haenszel random‐effects models were used to meta‐analyze the association of ACE exposure and MetS and its individual components.

**Results:**

A total of 16 papers (14 adult, 2 adolescent samples) met inclusion criteria, and 10 were eligible for meta‐analysis. There was a significant association between exposure of ≥ 1 ACE and MetS (odds ratio [OR] = 1.24, 95% confidence interval [CI] 1.18–1.29, *I* ^2^ = 82.2%, *p* < 0.001). Those with ≥ 3 ACEs vs. none had higher odds of MetS (OR = 1.43, 95% CI 1.32–1.55, *I* ^2^ = 63.1%, *p* = 0.019). Associations with ≥ 1 ACE were shown for hyperglycemia (OR = 1.27, 95% CI 1.20–1.33, *I* ^2^ = 88.1%, *p* < 0.001) and elevated blood pressure (OR 1.16, 95% CI 1.07–1.26, *I* ^2^ = 28.7%, *p* = 0.246). There were limited studies that examined the association between ACE and dyslipidemia and abdominal obesity. Some studies showed a stronger association of ACE and MetS among race/ethnic minorities compared with non‐Hispanic White individuals.

**Conclusions:**

Results show a dose–response relationship between ACE and MetS. These findings can inform the development of targeted interventions and policies to mitigate MetS risk among individuals with ACE exposure, particularly those from race/ethnic minority populations who may be at heightened risk.

**Abbreviations**
ACEs: adverse childhood experiences
CDC: Central for Disease Control and Prevention
CENTRAL: Cochrane Central Register of Controlled Trials databases
MetS: metabolic syndrome
NIH: National Institutes of Health
NIHR: National Institute for Health Research
OR: odds ratio
PRISMA: Systematic Reviews and Meta‐Analyses
US: United States

## 1 Introduction

According to the Center for Disease Control and Prevention (CDC), adverse childhood experiences (ACEs) are defined as potentially traumatic events that occurred in childhood (0–17 years) [1]. Typically, ACEs encompass exposure to abuse, neglect, or violence but can include other experiences that undermine a child's sense of safety, stability, and bonding such as food insecurity and parental incarceration [2]. The foundational 1998 Kaiser‐Permanente study by Felitti et al. identified seven core ACE domains: psychological abuse, physical abuse, sexual abuse, violence against the mother, household substance abuse, household mental illness or suicide, and household members imprisoned [3]. Subsequent studies have broadened the ACE framework to include additional ACE such as economic hardships, unsafe school environments, and bullying, all of which negatively affect health and well‐being [4].

ACEs are highly prevalent; 64% of United States (US) adults report that they have experienced at least one ACE and 17.3% report experiencing more than four ACEs [5]. ACEs have many associated adverse health outcomes including sexually transmitted infections, perinatal complications, adolescent pregnancy, and various chronic diseases [6]. The economic burden of ACE‐related health issues is estimated to be $748 billion annually in Bermuda, Canada, and the US combined [7]. Addressing ACEs and their health effects can potentially lead to significant savings and improvements in public health outcomes. Recent studies have highlighted the association between ACE and metabolic syndrome (MetS), a cluster of cardiometabolic disease risk factors that increase the likelihood of cardiovascular disease, type 2 diabetes mellitus (T2DM), and other chronic illnesses [3, 8, 9, 10, 11, 12, 13, 14, 15, 16, 17, 18, 19, 20, 21, 22].

Specifically, MetS encompasses a constellation of cardiometabolic disease risk factors including elevated blood pressure, blood sugar, and lipids, and abdominal obesity, which in turn are risk factors for chronic disease‐related morbidity and mortality [23]. The current recommended diagnostic criteria for MetS include the National Cholesterol Education Program's Adult Treatment Panel III (NCEP‐ATP III). For the NCEP‐ATP‐III, an individual indicated to have any of the following three traits would meet the MetS criteria: abdominal obesity (waist circumference > 40 in. in men or > 35 in. in women), serum triglycerides (≥ 150 mg/dL), high‐density lipoprotein cholesterol (< 40 mg/dL), fasting blood glucose level (≥ 100 mg/dL), and blood pressure (≥ 130/85 mmHg) [24].

A decade ago, the estimated US prevalence of MetS in adults was 37.6%, which increased to 41.8% in 2017–2018 [25]. While lifestyle risk factors such as diet, sleep, sedentary behavior, and physical activity, as well as genetic predisposition, have been associated with MetS [26], emerging evidence suggests that early‐life stressors, such as ACE, may also play a role in the development of MetS. Furthermore, a recent study by Comini et al. indicated that each MetS condition contributes unique, clinically important risk, and clustering of the conditions leads to higher disease burden. Comini and colleagues found that high fasting glucose, abdominal obesity, and reduced HDL‐cholesterol are each independently linked to conditions such as chronic kidney disease, suggesting the need to evaluate MetS components individually. This was especially important when identifying high‐risk patients and tailoring preventive strategies in clinical practice [27].

Understanding the association between ACE and MetS has significant implications for public health interventions and clinical practice. Early identification and prevention of individuals with a history of ACE may mitigate the long‐term health consequences associated with these experiences. The CDC states that 1.9 million heart disease cases could potentially be avoided through the prevention of ACE [1]. Because MetS is a precursor for cardiometabolic disease, determining the relationship between ACE and MetS could be a potential preventive strategy to reduce the burden of chronic disease. As a secondary objective, we will analyze each MetS component (elevated blood pressure, blood sugar, and lipids, and abdominal obesity) individually to determine which attributes may strongly drive the overall association with ACE. Currently, there is no comprehensive systematic review and meta‐analysis examining the association between ACE and MetS and its individual components in the scientific literature. Our study seeks to fill this crucial scientific gap by this updated review and meta‐analysis of this topic.

## 2 Methods

### 2.1 Study Protocol Registration

The study protocol is registered at PROSPERO (CRD42023487072), a database of prospectively registered systematic reviews funded by the National Institute for Health Research (NIHR) of the United Kingdom (https://www.crd.york.ac.uk/prospero/display_record.php?IDCRD42023487072). This report follows the Preferred Reporting Items for Systematic Reviews and Meta‐Analyses (PRISMA) reporting guidelines.

### 2.2 Search Strategy and Eligibility Criteria

Databases including PubMed, Embase, and Cochrane Central Register of Controlled Trials were used to search a combination of search terms including “adverse childhood experiences,” “traumatic childhood experience,” “childhood trauma,” or “early life stress,” and “insulin resistance,” “metabolic syndrome,” “lipid metabolism disorders,” “hypercholesterolemia,” “cardiometabolic syndrome,” “hypertension,” “central obesity,” or “hyperglycemia.” The search terms were modified for different databases. Studies published between 01/01/2000 and 02/01/2024 were included for initial screening. Duplicates were identified through Rayyan and excluded manually.

Studies were included if they met the following criteria: (1) original research articles with observational study designs (cross‐sectional, case–control, or cohort studies); (2) assessment of ACE defined as exposure to potentially traumatic events occurring before age 18, including but not limited to physical abuse, sexual abuse, emotional abuse, physical neglect, emotional neglect, and household dysfunction; (3) assessment of metabolic syndrome or its individual components (abdominal obesity, elevated blood pressure, dyslipidemia, hyperglycemia) as a primary outcome using established diagnostic criteria; and (4) examination of the association between ACE exposure and the presence of any individual component of MetS. Studies were excluded if they were protocols, not in the English language, case reports, non‐human studies, reviews, or stand‐alone abstracts.

### 2.3 Study Selection and Data Extraction

Title and abstracts of identified records were screened independently by two reviewers (JK and LX) before further full‐text screening. Differences between the two reviewers were assessed, reviewed, and resolved by a senior team member (SEM). We successfully obtained all articles meeting final inclusion criteria. The same two reviewers independently extracted data using a standardized form, resolving discrepancies through discussion. We organized extracted information into four categories: study characteristics (authors, publication year, country, design, sample size), participant demographics (age, sex, race/ethnicity), exposures (e.g., number and type of ACEs), and outcomes (MetS or its individual components).

### 2.4 Data Synthesis and Analysis

#### 2.4.1 Primary Exposure

The primary exposure across included studies was ACEs. We categorized studies based on the number of ACEs assessed, including any ACE exposure and the threshold of ≥ 3 (vs. < 3) ACEs, which we selected to capture a broader at‐risk population and enhance sensitivity for detecting associations.

#### 2.4.2 Primary Outcomes

The primary outcomes were the presence of any MetS components as well as individual components of MetS, specifically abdominal obesity, hyperglycemia, dyslipidemia, and elevated blood pressure. Both the overall presence of any MetS component and each individual component were examined in the included studies.

#### 2.4.3 Summary of Study Characteristics

From the 16 included papers, we extracted summary characteristics categorized into (1) the percentage of participants with ACE exposure, (2) primary exposure and outcomes examined, (3) type of ACEs assessed, and (4) MetS components measured. These characteristics, along with sex, mean age, and race/ethnicity of study populations, are summarized in Table 1.

**TABLE 1:** Study population characteristics.

Study	Country	Data source	Total *N*	Age	Female (%)	Race/ethnicity	Percentage of ACE population	Primary aim
**Cohort study**								
De Rubeis et al. [8]	Canada	Canadian Longitudinal Study on Aging	26,615	Not specified	75	White (94.5) Other (5.5)	66% had at least one ACE	Association between ACE and obesity in adulthood
Zhu et al. [4]	China	China Health and Retirement Longitudinal Study	9179	59 (SD not specified)	52	Chinese	85.6%	Association between ACE and diabetes
Tung et al. [9]	USA	US National Longitudinal Study of Adolescent to Adult Health	9629	37.8 (95% CI, 37.5–38.0 years)	50.3	White (71.4) American Indian or Alaska Native (0.8) Asian or Pacific Islander (3.5) Black or African American (14.9) Hispanic or Latino (8.8) Other (0.6)	14.1% experienced parental incarceration	Association between parental incarceration and long‐term health outcomes
Schuler et al. [10]	USA	Prospective data collection from a school	360	11.56 (0.57SD)	57.8	Non‐Hispanic White (3.9) Non‐Hispanic Black (76.7) Hispanic (16.4) Other (3.0)	75.3% experienced ≥ 1 ACE, 38.7% experienced ≥ 2 ACE, and 13.6% experienced ≥ 3 ACE	Association between threat and instability‐related exposures and cardiometabolic risk in adolescence
Fleischer et al. [22]	Germany	Cohort data from two health studies	2936	50 (SD not specified)	53.7	Not specified	Mean 7.7 child trauma score sum for women, and 7.8 for men	Association between childhood adversity and adult obesity
Allen et al. [12]	USA	The Oregon Health Insurance Experience	5851	Not specified	59.7	White, non‐Hispanic (73.9) Non‐White (25.8)	19.6% experience at least one ACE 13.2% experienced at least two ACEs 10.4% experienced at least three ACEs 35.6% experienced 4 or more ACEs	Association between ACEs and cardiovascular disease risk factors among low‐income uninsured adults
**Cross‐sectional study**								
Pretty et al. [13]	USA	School‐based data collection	1234	11.8 (SD 0.9)	55	Not specified	16% had at least 4 ACEs	Association between ACEs and early childhood risk factors for adult cardiovascular disease
Merrick et al. [2]	USA	Behavioral Risk Factor Surveillance System from 2015‐2017	144,017	Not specified	57.9	White (80.4) Black (8.4) American Indian/Alaska Native (2.0) Asian (1.2) Hispanic (6.0) Other (2.0)	60.9% experienced at least one type of ACE 15.6% had at least 4 or more ACEs	Association between ACEs and poor health and life outcomes in adulthood
Iqbal et al. [14]	USA	Medical records of a single hospital	110 children, and their parents	12.53 (SD 3.86)	43.3	White (88%) African American (5%) Others (7%)	27.9% among children and 49.0% among parents	Association of ACE and HbA1c, lipids and BMI z‐scores
Okwori et al. [15]	USA	Behavioral Risk Factor Surveillance System Data	5843 in TN 9630 in VA	Not specified	Not specified	Not specified	Not specified	Association between adverse childhood experiences and costs/burden of conditions
Campbell et al. [16]	USA	2011 Behavioral Risk Factor Surveillance System survey	48,526	Not specified	Not specified	White (85.24) Black (3.02) Hispanic (4.80) Other (6.94)	55.4% had at least one ACE, 13.4% had ≥ 4 ACEs	Association between ACE and risky behaviors and morbidity measures
Andrade et al. [17]	USA	Study done by an academic research institution in Florida	133	19.1 (1.3)	60.2	Hispanic (100)	19.% < 4ACEs 19.6% ≥ 4 ACEs	Association between ACE and cardiometabolic risk in Hispanic American adolescents
Maha Almueef [18]	Saudi Arabia	National survey	10,156	34.3 (SD 11)	47.9	Not specified	20.8% experienced childhood sexual abuse	Prevalence of different forms of CSA and its impact on chronic diseases, mental health disorders, health risk behaviors
El Mhamdi et al. [19]	Tunisia	A representative sample of adults living in the central eastern part of Tunisia	2120	36.3 (SD 12.7)	52.8	Not specified	80% experienced at least one early life adversity	Association between early life adversity and chronic health conditions
Clemens et al. [20]	Germany	A representative sample of Germany population	2510	48.4 (SD 18.2)	53.3	Not specified	30.3% experienced any form of child maltreatment	Association between child maltreatment and stressful life events during adulthood and cardiovascular problems
Campbell et al. [21]	USA	2011 Behavioral Risk Factor Surveillance System survey	48,526	Not specified	Not specified	White (85.24) Black (3.02) Hispanic (4.80) Other (6.94)	11.6% experienced sexual abuse	Association between sexual abuse in childhood and diabetes in adulthood

#### 2.4.4 Quantitative Synthesis

For meta‐analysis, we extracted odds ratios (ORs) and their 95% confidence intervals from studies reporting associations between ACE exposure (any ACEs and ≥ 3 ACEs) and the presence of any MetS components or individual MetS components. Meta‐analysis was performed using the metan command in STATA Version 15.1 [28], specifying the inverse variance approach for pooling the logit of the proportion. The pooled effect sizes and 95% confidence intervals are displayed in forest plots.

#### 2.4.5 Qualitative Synthesis

Only two studies reported the adjusted odds ratio (AOR) for the association of ACE and dyslipidemia (elevated triglyceride or low high‐density lipoprotein cholesterol concentrations), and one study reported the AOR for abdominal obesity; hence, meta‐analysis was not performed for those two components due to lack of statistical power. Instead, the association between ACE and these two components of MetS was summarized with available qualitative data.

#### 2.4.6 Power Analysis

Post hoc power analysis was conducted using the pwr package in R, accounting for the observed effect size, total sample size across studies, and between‐study heterogeneity. It shows that our meta‐analysis achieved > 99% power to detect the observed OR.

### 2.5 Risk of Bias Assessment

Study quality was assessed independently by two reviewers using the National Institutes of Health (NIH) standardized study quality assessment tools [29]. Disagreements were resolved through discussion. The NIH tools evaluate key methodological domains including study population definition and recruitment, exposure and outcome measurement validity, confounding variable control, statistical analysis appropriateness, and overall study design quality. Each study was rated as good, fair, or poor quality based on the tool's standardized criteria.

## 3 Results

**FIGURE 1:**
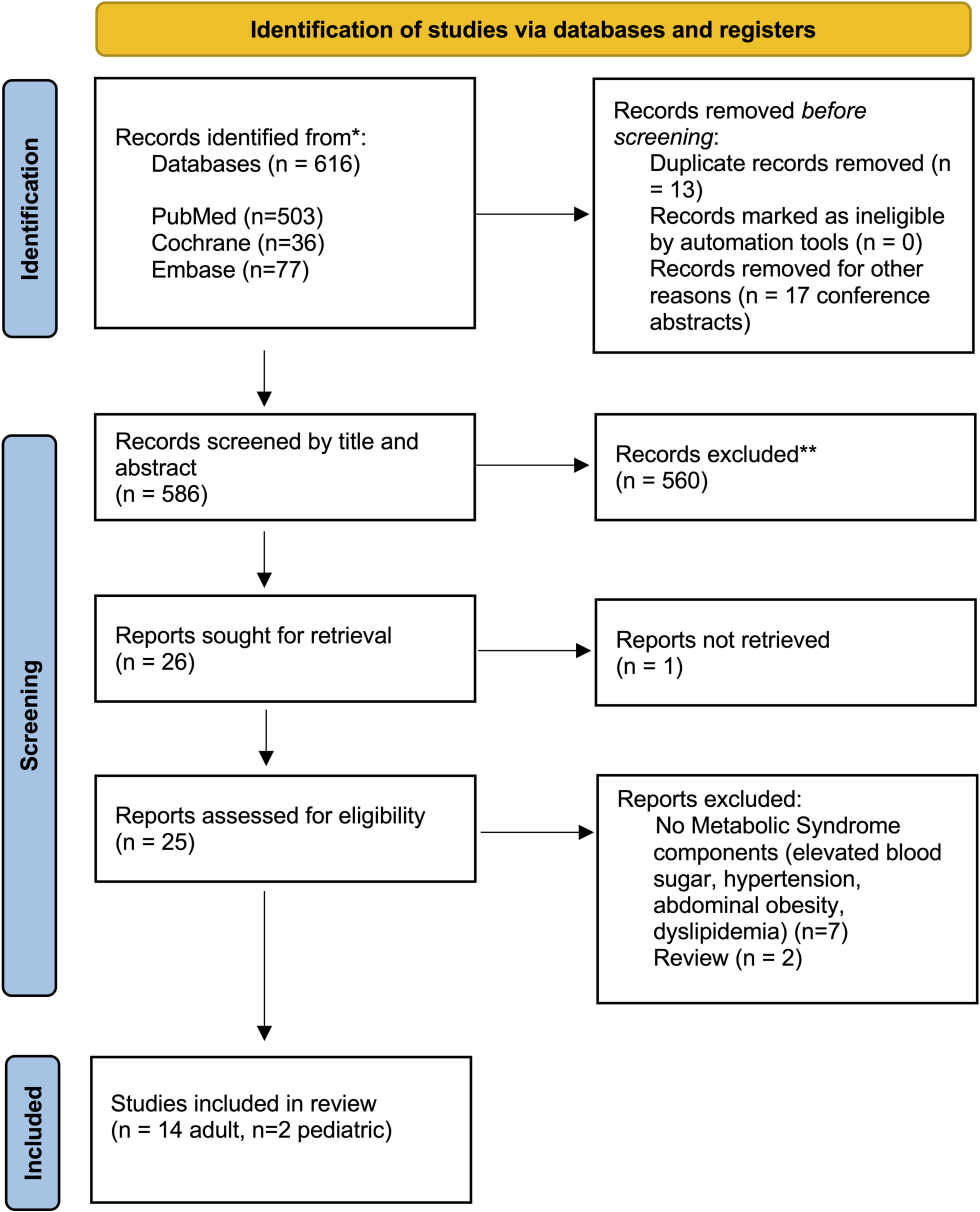
PRISM flow diagram. *Consider, if feasible to do so, reporting the number of records identified from each database or register searched (rather than the total number across all databases/registers). **If automation tools were used, indicate how many records were excluded by a human and how many were excluded by automation tools.

The study selection process applying PRISMA guidelines is demonstrated in Figure 1. The search first yielded a total of 616 records. Five duplicate records and 14 conference abstracts were removed, leaving 597 records for title and abstract screening. After exclusion of 532 abstracts that did not mention ACE and MetS (or components of MetS), a total of 65 full‐text articles were retrieved and reviewed for eligibility. The majority of these (*n* = 39) were excluded due to no information on the association between ACE and MetS. Finally, 16 publications met the prespecified inclusion criteria and were included in the systematic review.

### 3.1 Study Characteristics

The majority of studies (*n* = 10) were conducted in the US, with other countries such as Germany (*n* = 2), Canada (*n* = 1), China (*n* = 1), Saudi Arabia (*n* = 1), and Tunisia (*n* = 1). Cross‐sectional studies constituted the majority of study designs with 10 out of 16 studies, alongside three prospective and three retrospective cohort studies. Sample sizes ranged from 110 to 144,017 participants, and the total sample size across all included studies was 278,649 participants.

Eleven out of 16 included studies investigated more than eight ACEs and were primarily interested in cumulative ACE scores with the upper limit being 15 different ACEs. These studies commonly included experiences such as physical, emotional, and sexual abuse; physical and emotional neglect; and various forms of household dysfunction, including parental substance use, mental illness, incarceration, domestic violence, and parental separation or death. Additional ACE exposures such as economic hardship, bullying, housing instability, and exposure to unsafe school environments were also assessed. Five included studies focused on less than eight ACEs, with four of the five studies being focused on a singular ACE experience such as child maltreatment, parental incarceration, child sexual abuse, or specific threat and instability‐related experiences. The vast majority of studies (nine out of 10) utilized retrospective recall methods. Many of the US studies used self‐report or parental‐report questionnaires such as the Behavorial Risk Factor Surveillance System, Childhood Trauma Questionnaire, and the original CDC‐Kaiser ACE study items. Studies done outside of the US commonly utilized the ACE‐International Questionnaire, another retrospective self‐report method. Only one study, conducted by Schuler et al. assessed ACE prospectively by collecting annual trauma and instability data from youth over a 3‐year period.

Among the included studies, the majority of studies examined multiple outcomes related to cardiometabolic and general health. Common outcomes included body mass index (BMI), waist circumference, body fat percentage, blood pressure, cholesterol level, insulin sensitivity, glycemic control, and the presence of chronic conditions such as diabetes, hypertension, coronary heart disease, respiratory disease, and mental health disorders. Some studies also investigated behavioral and lifestyle outcomes such as smoking, alcohol use, physical activity, and risky sexual behavior. Because the scope of our study is on MetS outcomes, we found that out of our included studies, 10 examined hyperglycemia, 3 explored abdominal obesity, 3 investigated dyslipidemia, and 6 studied elevated blood pressure.

Despite the diversity of outcomes assessed, none of the studies included a computed MetS composite score. Instead, outcomes were typically reported individually. In nearly all the studies, the outcome data were not mutually exclusive as individuals were represented in multiple outcome categories simultaneously. Similarly, ACE exposures also often overlapped as participants were allowed to usually report more than one ACE. These factors contributed to the non‐mutual exclusivity in the reporting of these studies.

Most studies were conducted in adults, with a predominantly female population (43.3%–75%), and two studies included children. Average participant ages spanned from 11.56 to 59 years. Race or ethnicity was specified in 10 studies, with seven predominantly featuring non‐Hispanic White participants (ranging from 71.4% to 95%). One study exhibited a more racially/ethnically diverse sample, with non‐Hispanic Black participants comprising 76.7% of the cohort, while the remaining two studies focused on Chinese and Hispanic samples.

Out of the 16 publications, 15 specified the percentage of ACE within the overall population. Studies concentrating on a single ACE, such as sexual abuse and parental incarceration, reported lower prevalence estimates ranging from 11.6% to 30.3%. Conversely, those exploring a broad range of ACE (8–15 different types) reported that 19.6% to 80% of participants experienced at least one ACE. Furthermore, five studies focusing on a wide array of ACE reported that 13.4% to 35.6% of participants had encountered four or more ACE [2, 12, 13, 16, 17].

### 3.2 Association of ACE and MetS

**FIGURE 2:**
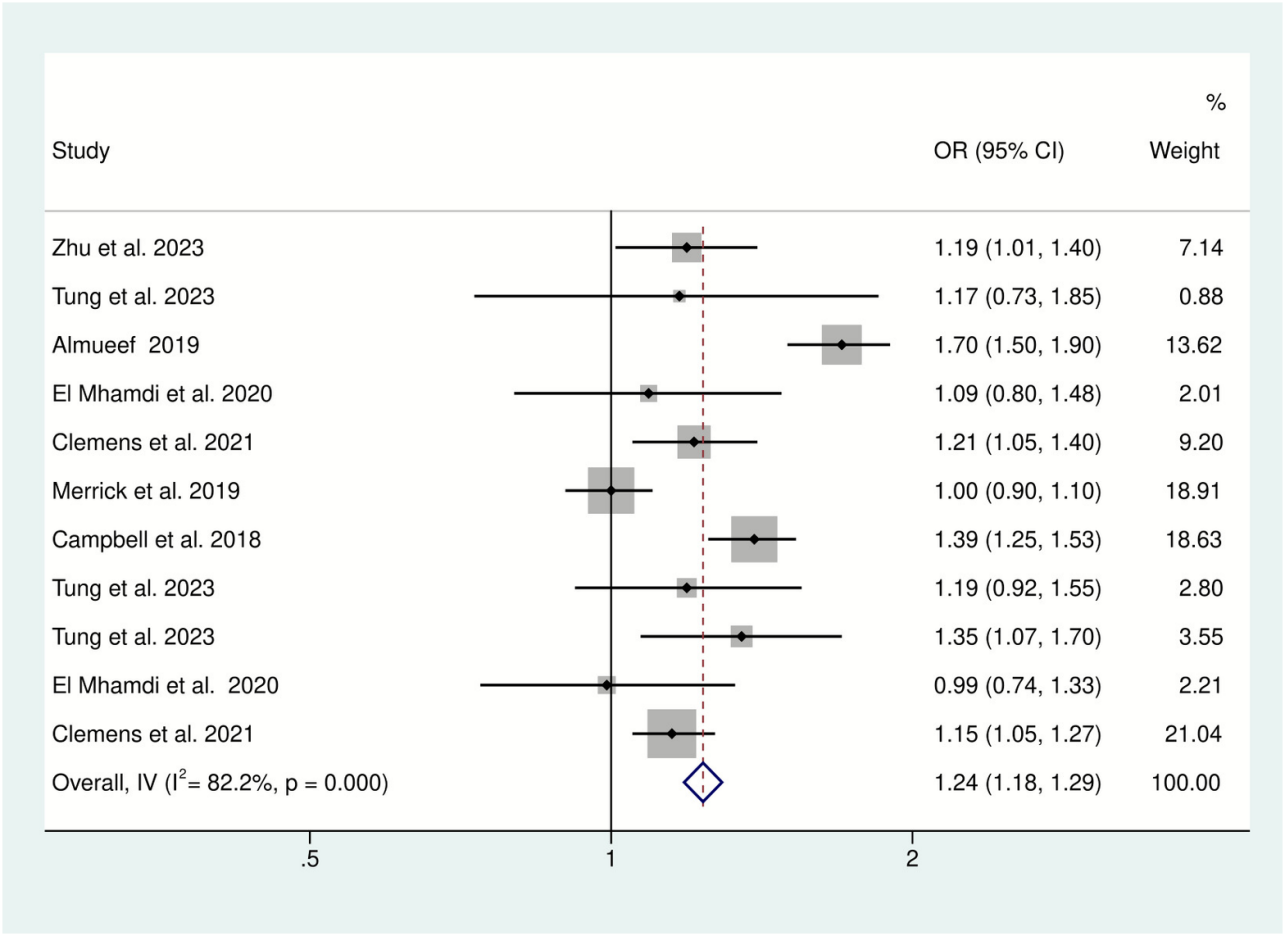
Exposure to at least one ACE and outcome of MetS.

**FIGURE 3:**
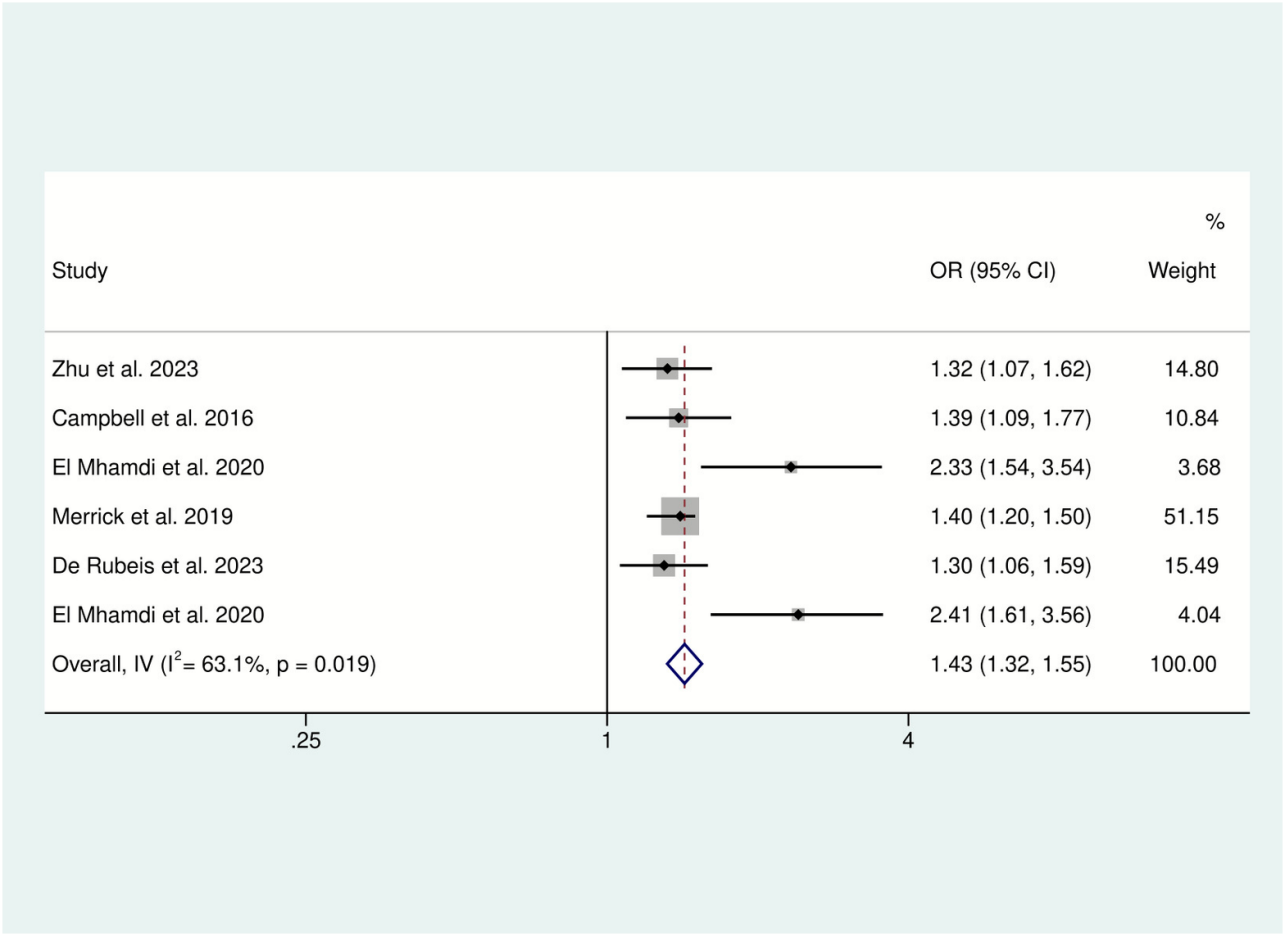
Exposure to at least three ACE and outcome of MetS. *Note:* The largest ACE category captured across all studies was ≥ 4 ACE (*n* = 18,350; Merrick et al. 2019).

A meta‐analysis of 10 studies was conducted to investigate the OR of the association between experiencing one ACE and the presence of any components of MetS [2, 4, 9, 16, 18, 19, 20]. Figure 2, summarizing the pooled analysis, illustrates that the OR was 1.24 (95% confidence interval [CI] 1.18–1.29, *I* ^2^ = 82.2%, *p* < 0.001), indicating individuals who were exposed to at least one ACE in childhood were 24% more likely to have MetS in later life. Furthermore, we also investigated the association between experiencing three or more ACE and MetS [2, 4, 8, 16, 19]. Results showed that individuals who experienced three or more ACE had 1.4 times higher odds of experiencing MetS (OR = 1.43, 95% CI 1.32–1.55, *I* ^2^ = 63.1%, *p* = 0.019) (Figure 3).

### 3.3 Association of ACE and Type 2 Diabetes (T2DM)

Furthermore, a meta‐analysis was conducted to explore the association between experiencing at least one ACE and T2DM [2, 4, 9, 16, 17, 18, 19, 20]. (Figure S1). Of the 16 publications included in this systematic review, seven studies focused on the association between ACE and T2DM (Table 2). There was a significant positive association between at least one ACE and T2DM with an overall OR of 1.27 (95% CI 1.20–1.33, *I* ^2^ = 88.1%, *p* < 0.001). We also compared the association between three or more ACE and T2DM (Figure S2) [2, 4, 16, 19]. The results showed that the odds of having T2DM are 1.42 (95% CI 1.30–1.55, *I* ^2^ = 50.0%, *p* = 0.111) times higher in individuals who experienced three or more ACE than those who experienced less than three ACE. These findings suggest that the more ACE experienced, the higher the odds of experiencing T2DM.

**TABLE 2:** Summary of association between adverse childhood experiences and elevated blood glucose levels.

Study	Type of ACE	Primary association of ACE and elevated blood sugar levels
Iqbal et al. [14]	9 different ACEs considered: Jail Racial discrimination Parent death Divorce/separation Witness/victim of violence in the neighborhood Domestic violence in family Mental illness Financial difficulties Substance abuse	Children with 3 or more ACEs vs. < 3: *β* = 0.63 (95% CI 0.11–1.15) increase in HbA1C levels Parents with 4 or more ACEs vs. < 4: *β* = 0.87 (95% CI 0.11–1.63) increase in HbA1C levels
Zhu et al. [4]	15 different ACEs considered: Emotional neglect Emotional abuse Physical abuse Family members with mental illness Family members with substance abuse Incarceration Domestic violence Poor parental relationship Parental divorce Parental disability Parental death Economic adversity Unsafe school environment Bullying Bad community environment	Any ACE vs. no ACE: OR = 1.19 (95% CI 1.01–1.40) for diabetes 3 ACEs or more vs. no ACE: OR = 1.32 (95% CI 1.07–1.62) for diabetes 4 or more ACEs vs. no ACE: OR = 1.29 (95% CI 1.07–1.56) for diabetes ACE subtypes: Family members with Substance Abuse: OR = 1.23 (95% CI 1.08–1.41) Emotional Abuse: OR = 1.25 (95% CI 1.12–1.46) Poor rental relationship: OR = 1.25 (95% CI 1.09–1.43)
Campbell et al. [16]	11 different ACEs considered: Lived with anyone who was depressed, mentally ill, or suicidal Lived with anyone who was a problem drinker or alcoholic Lived with anyone who used illegal drugs or abused prescription drugs Lived with anyone who served time in a correctional facility Experienced parental separation or divorce Witnessed parents or adults in the home slap, hit, kick, punch, or beat each other Being slapped, hit, kicked, punched, or beat by parents or adults in the home Being sworn at, insulted, or put down by parents or adults in the home Being touched sexually by adult or anyone 5 years older than respondent Being made to touch sexually at adult or anyone 5 years older than respondent; and Being forced to have sex with an adult or anyone 5 years older than respondent	4 or more ACE vs. no ACEs: AOR of 1.39 (95% 1.09–1.77) for diabetes
Tung et al. [9]	1 ACE considered: Parental incarceration	Parental incarceration vs. none: AOR of 1.17 (95% CI 0.73–1.85) for Diabetes
Okwori et al. [15]	11 ACEs considered: Sworn at/insulted Physically hurt Forced to touch sexually Forced touch sexually Forced sex Witnessed parents beat each other up Lived with parent/guardian who was depressed Lived with alcoholics Parent/Guardian who abused drugs Parent/guardian imprisoned Parents divorced	17% had diabetes with one of more ACE
Maha Almueef [18]	1 ACE considered: Child sexual abuse	Child sexual abuse vs. none: AOR of 1.7 for diabetes (95% CI 1.5–1.9)
El Mhamdi et al. [19]	Intrafamilial ELA (ELA experienced in the home) Conflictual relationship to parents/caregivers Neglect Household dysfunction Physical abuse Sexual abuse Social ELA (ELA experienced in society) Peer violence Witnessing community violence Exposure to collective violence	Diabetes: 1 social ACE vs. none: AOR of 1.09 (95% CI 0.80–1.48) 2 social ACE vs. none: AOR of 1.86 (95% CI 1.39–2.48) 3 social ACE vs. none: AOR of 2.33 (95% CI 1.54–3.54)
Clemens et al. [20]	Child maltreatment	Child Maltreatment vs. none: AOR of 1.21 (95% CI 1.05–1.40) for Diabetes
Merrick et al. [2]	8 ACEs considered: 3 types of abuse: Physical abuse Emotional abuse Sexual abuse 5 types of household challenges: Household member substance misuse Incarceration Mental illness Parental divorce Witnessing intimate partner violence	Diabetes: 1 ACE vs. none: AOR of 1.0 (95% CI 0.9–1.1) 2–3 ACEs vs. none: AOR of 1.1 (95% CI 1.1–1.2) 4 or more ACEs vs. none: AOR of 1.4 (95% CI 1.2–1.5)
Campbell et al. [21]	1 ACE considered: Sexual abuse	Sexual Abuse vs. none: OR of 1.39 (95% CI 1.25–1.53) for Diabetes

### 3.4 Association of ACE and Elevated Blood Pressure

Three studies were available to investigate the association between experiencing one ACE and elevated blood pressure [9, 19, 20] (Table 3). The overall OR for the association between at least one ACE and elevated blood pressure was 1.16 (95% CI 1.07–1.26, *I* ^2^ = 28.7%, *p* = 0.246). (Figure S3).

**TABLE 3:** Summary of association between adverse childhood experiences and hypertension.

Study	Type of ACE	Primary association of ACE and hypertension
Tung et al. [9]	1 ACE considered Parental incarceration	Parental incarceration vs. none: AOR of 1.35 (95% CI 1.07–1.70) for hypertension
Okwori et al. [15]	11 ACEs considered Sworn at/insulted Physically hurt Forced to touch sexually Forced touch sexually Forced sex Witnessed parents beat each other up Lived with parent/guardian who was depressed Lived with alcoholics Parent/guardian who abused drugs Parent/guardian imprisoned Parents divorced	*N* = 5843 for Tennessee Prevalence of hypertension: Sworn at/insulted—38.49% Physically hurt—41.72% Forced to touch sexually—45.34% Forced touch sexually—46.08% Forced sex—48.79% Witnessed parents beat each other up—41.78% Lived with parent/guardian who was depressed—38.04% Lived with alcoholics—45.04% Parent/guardian who abused drugs—36.69% Parent/guardian imprisoned—40.56% Parents divorced—34.58% *N* = 9640 for Virginia Prevalence of hypertension sworn at/insulted—38.10 Physically hurt—42.31% Forced to touch sexually—40.91% Forced touch sexually—42.64% Forced sex—43.68% Witnessed parents beat each other up—42.27% Lived with parent/guardian who was depressed—35.42% Lived with alcoholics—44.37% Parent/guardian who abused drugs—35.35% Parent/guardian Imprisoned—36.34% Parents divorced—38.55%
El Mhamdi et al. [19]	Intrafamilial ELA (ELA experienced in the home) Conflictual relationship to parents/caregivers Neglect Household dysfunction Physical Abuse Sexual Abuse Social ELA (ELA experienced in society) Peer violence Witnessing community violence Exposure to collective violence	Hypertension: 1 social ACE vs. none: AOR of 0.99 (0.74–1.33) 2 social ACEs vs. none: AOR of 1.61 (1.21–2.14) 3 social ACEs vs. none AOR of 2.41 (1.61–3.56)
Clemens et al. [20]	Child maltreatment	Child maltreatment vs. none: AOR of 1.15 (95% CI 1.05–1.27) (for hypertension)
Allen et al. [12]	10 ACEs considered 3 types of abuse: Physical abuse Emotional abuse Sexual abuse 2 types of neglect: Emotional neglect Physical neglect 5 types of household dysfunction: Household member substance misuse Incarceration Mental illness Parental divorce Witnessing intimate partner violence	With every additional ACE added, the probability of having a prestudy diagnosis of hypertension went up by 1 percentage point (0.002)
Andrade et al. [17]	Household alcohol/street drug misuse Parental divorce or separation Household mental illness/suicide attempt Household member incarcerated Witnessed domestic violence Physical abuse Physical neglect Emotional abuse Emotional neglect Sexual abuse Bullying (physical, verbal, social, cyber)	4 or more ACEs vs. < 4: *β* = 0.037 (SE 0.018, *p* = 0.05) for diastolic blood pressure

### 3.5 Association of ACE and Abdominal Obesity

Out of the 16 studies included in this review, three examined the association between ACE and abdominal obesity [8, 11, 13]. However, due to the limited availability of data on ORs across studies, a meta‐analysis could not be conducted. Table 4 shows that De Rubeis et al. [8] reported that men and women with four or more ACE, compared with those with none, had 1.3 times higher odds of having abdominal obesity (95% CI 1.15–1.47). Pretty et al. [13] demonstrated a significant increase in waist circumference (*β* = 3.6, *p* < 0.01) among individuals who experienced four or more ACE. Fleischer et al. [22] revealed a relatively strong correlation between childhood trauma score and waist‐to‐height ratio (WHtR) in both women (*β* = 0.074, *p* = 0.004) and men (*β* = 0.133, *p* < 0.001). Among these three papers, obesity outcomes encompassed both central measures (e.g., waist circumference or waist‐to‐height ratio) and general obesity defined by BMI.

**TABLE 4:** Summary of association between adverse childhood experiences and excess abdominal weight.

Study	Type of ACE	Primary association of ACE and excess abdominal weight
De Rubeis et al. [8]	8 different ACEs considered: Physical abuse Sexual abuse Emotional abuse Neglect Exposure to intimate partner violence Death of a parent Parental divorce/separation Living with a family member with mental health problems before the age of 18.	4 or more ACEs vs. no ACE: AOR of 1.3 (95% CI 1.06–1.59) in waist circumference in men and AOR of 1.29 (95% CI 1.10–1.51) in waist circumference in women
Pretty et al. [13]	12 different ACEs considered: Death of family member (not parent) Loss a pet that they really cared for (died, killed, lost) Serious illness or injury in the family Conflict or serious argument between parents Divorce or separation of parents At least one night stay in a hospital Serious illness or injury Separation from parents Badly frightened or attacked by an animal Saw someone get badly hurt or die suddenly Family member or residence was robbed Death of a parent	4 or more ACEs vs. < 4: *β* = 3.6 (95% CI 1.8–5.3) increase in waist circumference
Fleischer et al. [22]	Childhood trauma screening included: Physical abuse/neglect Emotional abuse/neglect	Childhood Trauma Score was significantly associated with WHtR in women (beta = 0.074, *p* = 0.004) and men (beta = 0.133, *p* < 0.001)

### 3.6 Association of ACE and Dyslipidemia

Two out of 16 papers reported results on the association of ACE and dyslipidemia [9, 10]. Due to the limited data available, a meta‐analysis could not be conducted. However, Table 5 shows that Tung et al. [9] found a moderate association between ACE and the presence of hyperlipidemia (OR = 1.19). A different study by Schueler et al. [10] demonstrated that individuals with two or more ACE, compared with those with none, exhibited an increased level of triglycerides (*β* = 0.14, SE = 0.002, 95% CI 0.002, 0.01, *p* = 0.004). Schueler et al. [10] also found that individuals with three or more ACE, compared with none, had lower levels of high‐density lipoprotein (HDL) cholesterol (*β* = −0.88, SE = 0.36, 95% CI −1.59 to −0.18, *p* = 0.01).

**TABLE 5:** Summary of association between adverse childhood experiences and dyslipidemia.

Study	Type of ACE	Primary association of ACE and hypertriglyceridemia
Tung et al. [9]	1 ACE considered—Parental incarceration	Parental incarceration vs. none: AOR of 1.19 (95% CI 0.92–1.55) for hyperlipidemia
Schuler et al. [10]	Several ACEs considered A. Unexpected events that harm, victimize, or threaten children Arrested by police Crime victim Sudden loss of parent or close relationship B. Lack of or unstable access to necessary basic physical and social resources such as unstable housing Multiple relocations Uncertainty of housing stability Inconsistent access to food Irregular access to basic utilities	2 or more ACEs vs. none: Increasing slope of triglycerides (b = 0.007, SE = 0.002, *β* = 0.14 [95% CI 0.002, 0.01], *p* = 0.00)4
Schuler et al. [10]	Several ACEs considered A. Unexpected events that harm, victimize, or threaten children Arrested by police Crime victim Sudden loss of parent or close relationship B. Lack of or unstable access to necessary basic physical and social resources such as unstable housing Multiple relocations Uncertainty of housing stability Inconsistent access to food Irregular access to basic utilities	3 or more ACEs vs. none: Lower HDL (b = −0.88, *p* = 0.1)

## 4 Discussion

To our knowledge, this study is the first systematic review and meta‐analysis examining the association between ACE and MetS. Through an extensive search strategy and meticulous data extraction process, 16 relevant papers were identified for inclusion in this systematic review, with 10 meeting the eligibility criteria for meta‐analysis. The collective body of evidence synthesized in this review underscores a significant association between ACE and MetS. The main finding in this meta‐analysis reveals a consistent and statistically significant positive association between ACE exposure and MetS, regardless of the number of ACE experienced. Additionally, this study examined the association of ACE with specific components of MetS. Notable results showed that patients who experienced ACE had 20% higher odds of T2DM or hypertension. Interestingly, the analysis also showed that the OR for developing MetS is higher for participants experiencing three or more ACE compared with those who experienced one ACE, suggesting that there is a dose–response relationship between the number of ACE experienced and risk of MetS, emphasizing the enduring impact of early‐life adversity on cardiometabolic health outcomes. Analysis also showed a stronger association of ACE and MetS among race/ethnic minorities compared with non‐Hispanic White individuals. Results here suggest that incorporation of ACE screenings into clinical workflows, such as during patient‐history intake forms or annual wellness checks, may help identify patients at risk for MetS, T2DM and heart disease, and especially among those who identify as a race/ethnic minority

Our findings underscore the need for population‐level strategies that reduce the incidence of ACEs in the first place. At the clinical level, several US jurisdictions now embed routine ACE screening within primary‐care encounters and chronic‐disease management visits [30]. A leading example is California's state‐sponsored ACEs Aware initiative, which provides billing codes, on‐line training modules, and decision‐support tools to help clinicians translate positive screens into trauma‐informed care and referrals to outpatient clinics [31]. As of the June 2025 Data report, more than 4.2 million ACE screenings had been completed by Medi‐Cal providers [32]. Many of these providers focus on leveraging evidence‐based services—such as Diabetes Prevention Programs, nutritional counseling, and stress–management coaching—to improve long‐term outcomes [33]. Notably, the initiative has significantly increased identification of children eligible for Pediatric Enhanced Care Management, facilitating earlier multidisciplinary support [34].

While one prior review has explored the association between ACEs and MetS specifically within specific populations [35], to our knowledge, this is the first systematic review to examine the relationship between ACEs and MetS in the general population. This prior study, conducted by Balaji et al. in 2023, explored the correlation between ACE and MetS in individuals with severe mental illness [35]. Their review, encompassing 20 studies, concluded that there was no significant association between ACE and either the components of MetS or MetS. Notably, they utilized obesity and BMI as outcomes for MetS, despite obesity not being a formal component of MetS. In contrast, our study reveals a significant association between ACE and MetS. This suggests a key discrepancy rooted in different factors such as including obesity as an indicator for MetS or the population characteristics that may account for the difference observed. A separate review by Zhu et al. in 2022 investigated the link between ACE and the risk of T2DM, encompassing a total of 49 studies [36]. Their analysis reported an OR of 1.22 for individuals with any ACE, rising to 1.44 for those with four or more ACE. These findings closely mirror our systematic review regarding the overall ORs indicative of the association between ACE and MetS. Further comparisons with published literature proved challenging, given the absence of a comprehensive systematic review and meta‐analysis specifically addressing the relationship between ACE and MetS prior to our study.

Furthermore, a notable research gap highlighting race/ethnicity group disparities was also identified in this review. Race and ethnicity were explicitly specified in 10 of the studies reviewed, with seven predominantly featuring non‐Hispanic White participants (constituting between 71.4% and 95% of the sample) [2, 8, 9, 12, 14, 16, 21]. Conversely, one study exhibited a more ethnically diverse cohort, with non‐Hispanic Black participants representing 76.7% of the sample [10]. When comparing data from existing literature, this finding is concerning given children from different racial and ethnic backgrounds experience ACE disparately. In the United States, 61% of non‐Hispanic Black children and 51% of Hispanic children have been exposed to at least one ACE. This starkly contrasts with lower rates of 40% among non‐Hispanic White children and 23% among non‐Hispanic Asian children that have been exposed to at least one ACE [37]. This disparity is reflected throughout our systematic review as well, as evidenced by Schuler et al.'s study reporting 75.3% of individuals (76.7% non‐Hispanic Black and 16.4% Hispanic) experienced at least one ACE [10]. Schuler et al.'s study can be compared with Allen et al.'s study that reported only 19.6% of individuals (73.9% non‐Hispanic White and 25.8% non‐White) experienced at least one ACE [12]. Additionally, a study focused on the racial and ethnic differences in clusters of ACE highlights that most non‐Hispanic White adolescents belong to a low adversity class, while most non‐Hispanic Black and Hispanic adolescents are categorized into a group characterized by high adversity relative to socioeconomic status (SES) and parental incarceration [38]. Consequently, it is highly recommended that future research prioritizes the inclusion of more racially and ethnically diverse samples to ensure a comprehensive understanding of the association between ACE and MetS.

Our systematic review highlights a noticeable lack of studies investigating the link between ACE and dyslipidemia, as well as abdominal obesity. While previous studies have explored the association between ACE, T2DM, general obesity, and elevated blood pressure, the limited published data on dyslipidemia is particularly concerning due to the substantial burden of cardiovascular diseases associated with lipid abnormalities and abdominal obesity [39, 40]. Tsao et al.'s study emphasizes this concern by reporting that 10% of adults aged 20 or older had total cholesterol levels exceeding 240 mg/dL, and 17% exhibited HDL cholesterol levels below 40 mg/dL [41]. Moreover, data from Sun et al. revealed that a staggering 53.13% of the US population is affected by abdominal obesity [42]. Within our systematic review, Tung et al. indicated that exposure to an ACE is significantly associated with hyperlipidemia [9], and two or more ACE are significantly associated with an increasing level of triglycerides [10]. Additionally, our review highlights three studies linking ACE exposure to abdominal obesity [8, 11, 13], with one notably reporting a 30% increased odds of abdominal obesity among individuals experiencing four or more ACE [8]. Conclusively, our study also emphasizes the paucity of available data for further analysis of dyslipidemia and abdominal obesity, emphasizing the imperative for future research to address these critical outcomes with ACE.

A possible mechanism for the association between ACE and MetS could be the role in promoting dysregulation in key metabolic pathways. Published literature emphasizes that ACE can lead to chronic stress, triggering the release of cortisol, a primary stress hormone [43]. Prolonged elevation of cortisol levels can disrupt metabolic processes, such as glucose metabolism and insulin sensitivity, contributing to the development of hyperglycemia, type 2 diabetes, and MetS [44]. Additionally, cortisol can influence appetite regulation and promote visceral fat deposition, further exacerbating MetS factors such as obesity and dyslipidemia [45]. Thus, the interplay between ACE, cortisol dysregulation, and metabolic disturbances may provide a plausible explanation for the observed association between ACE and MetS.

### 4.1 Limitations and Strengths

There are some limitations to consider when interpreting our findings. First, significant heterogeneity was observed across multiple meta‐analyses, which may be attributed to variations in study characteristics, such as the study population, types of ACE studied (e.g., maltreatment vs. family dysfunction), and study designs employed. While conducting formal sensitivity analyses to explore potential sources of heterogeneity, such as grouping studies by ACE type, was considered, it was not feasible due to the small number of eligible studies (*n* = 10) and inconsistent reporting of ACE domains. Nonetheless, this remains an important area for future investigation as more studies become available and reporting becomes more standardized. Another potential limitation is the presence of publication bias, wherein studies with stronger findings are more likely to be published, thus skewing the overall results. Additionally, the majority of included studies were cross‐sectional, which limits the ability to establish temporality and may have contributed to variability in effect estimates. Lastly, another limitation of this study is the inclusion of papers focusing on T2DM rather than hyperglycemia, which is the actual component of MetS. This decision was due to the limited literature specifically investigating hyperglycemia. Given the close association between T2DM and hyperglycemia, we opted to incorporate studies on T2DM to provide insights into the broader cardiometabolic implications. Although studies have shown that ACE can be accurately recalled throughout adulthood [46], a limitation of our study can be that many of our included studies utilized retrospective assessment methods. This is expected as generally ACE data are typically gathered through retrospective parental or self‐reports and may be influenced by recall bias.

However, despite these limitations, our study represents the first systematic review and meta‐analysis to comprehensively examine the association between ACE and MetS and its components. Our study aims to contribute valuable insights to the existing literature and lay the groundwork for further research in this important area of study.

### 4.2 Conclusion

In conclusion, the evidence from our systematic review and meta‐analysis suggests an association between ACE and MetS, as well as its individual components. The more ACE experienced by individuals, the higher the likelihood of having MetS in later life, indicating a dose–response relationship between ACE and MetS. However, while previous reviews have examined related associations, including those in specific subpopulations, our review highlights ongoing research gaps, particularly the need for more studies investigating the association between ACE and dyslipidemia as well as abdominal obesity. Additionally, the lack of racial and ethnic diversity in study samples highlights the need for future research to prioritize the inclusion of diverse populations for a complete understanding. Ultimately, our study underscores the influence of early‐life adversity on metabolic health and emphasizes the importance of targeted interventions aimed at mitigating the long‐term health consequences of childhood trauma.

## Author Contributions

Joohan Kim conceptualized and designed the study, carried out the initial analyses, drafted the initial manuscript, and critically reviewed and revised the manuscript. Dr. Luyu Xie conceptualized and designed the study, reviewed analyses, drafted the initial manuscript, and critically reviewed and revised the manuscript. Dr. Sarah E. Messiah, Dr. Jaime P. Almandoz, and Dr. Alejandra Fernandez critically reviewed and revised the manuscript for important clinical and other content. All authors approved the final manuscript as submitted and agreed to be accountable for all aspects of the work.

## Funding

This study was supported by the National Institute of Child Health and Human Development (grant number R21HD105129), the National Institute on Minority Health and Health Disparities (grant number R01MD011686), and the National Heart, Lung, and Blood Institute (K01HL166439‐01A1). The NIH had no role in the design and conduct of the study.

## Conflicts of Interest

Dr. Messiah reported receiving grants from the National Institute on Minority Health and Health Disparities (NIMHD) and the National Institute of Child Health and Human Development (NICHD) during the conduct of this study. Dr. Xie reported receiving funding from NIMHD during the conduct of this study. Dr. Fernandez reported receiving funding from NHLBI during the conduct of this study. Dr. Almandoz reported receiving funding from consulting fees from Novo Nordisk, Boehringer Ingelheim, and Eli Lilly Company, in addition to funding from NIMHD during the conduct of this study.

## Supporting information


**Figure S1:** Exposure to at least 1 ACE and Diabetes.
**Figure S2:** Exposure to 3 or more ACEs and Diabetes.
**Figure S3:** One ACE and Hypertension.

## Data Availability

Data sharing is not applicable to this article as no datasets were generated or analyzed during the current study.
