# Supplementary material for: Association of Adverse Childhood Experiences and Metabolic Syndrome: A Systematic Review and Meta‐Analysis
Source: Obes Rev. 2026 Jan 27;27(7):e70095. doi: 10.1111/obr.70095 (PMC13243344; doi:10.1111/obr.70095)

Association of Adverse Childhood Experiences and Metabolic Syndrome: A Systematic Review and Meta-analysis

Joohan Kim[a,b#], Luyu Xie, PharmD, PhD[b,c#], Alejandra Fernandez, PhD[b,d], Jaime P. Almandoz, MD, MBA, FTOS[e], Sarah E. Messiah, PhD, MPH, FTOS[b,c,e]

Affiliations: [a]Texas A&M University, College Station, TX, USA; [b]Center for Pediatric Population Health, University of Texas Health Science Center at Houston (UTHealth) School of Public Health, Dallas, TX, USA; [c]Department of Epidemiology, Human Genetics and Environmental Sciences, UTHealth School of Public Health, Dallas, TX, USA; [d] Department of Health Promotion and Behavioral Sciences, UTHealth School of Public Health, Dallas, TX, USA; [e]Department of Internal Medicine, Division of Endocrinology, University of Texas Southwestern Medical Center, Dallas, TX, USA; [e]Department of Pediatrics, McGovern Medical School, Houston, TX USA

[#]Joohan Kim and Luyu Xie contributed equally

Address correspondence to:
Sarah E. Messiah
University of Texas Health Science Center at Houston
School of Public Health in Dallas
Center for Pediatric Population Health
2777 North Stemmons Freeway, Suite 8400
Dallas, TX 75390
Tel: 972-546-2919
Fax: 214-351-7006
Email: Sarah.E.Messiah@uth.tmc.edu

Supplemental Figure 1. Exposure to at least 1 ACE and Outcome of Diabetes

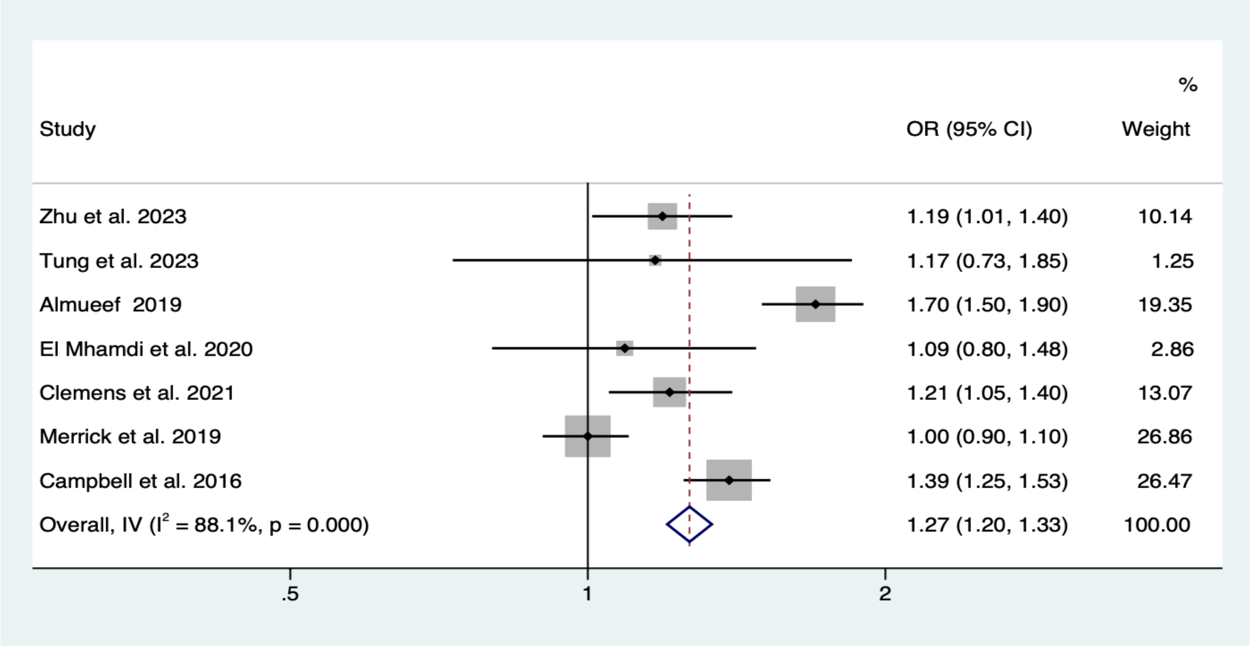

Supplemental Figure 2. Exposure to 3 or more ACEs and Outcome of Diabetes

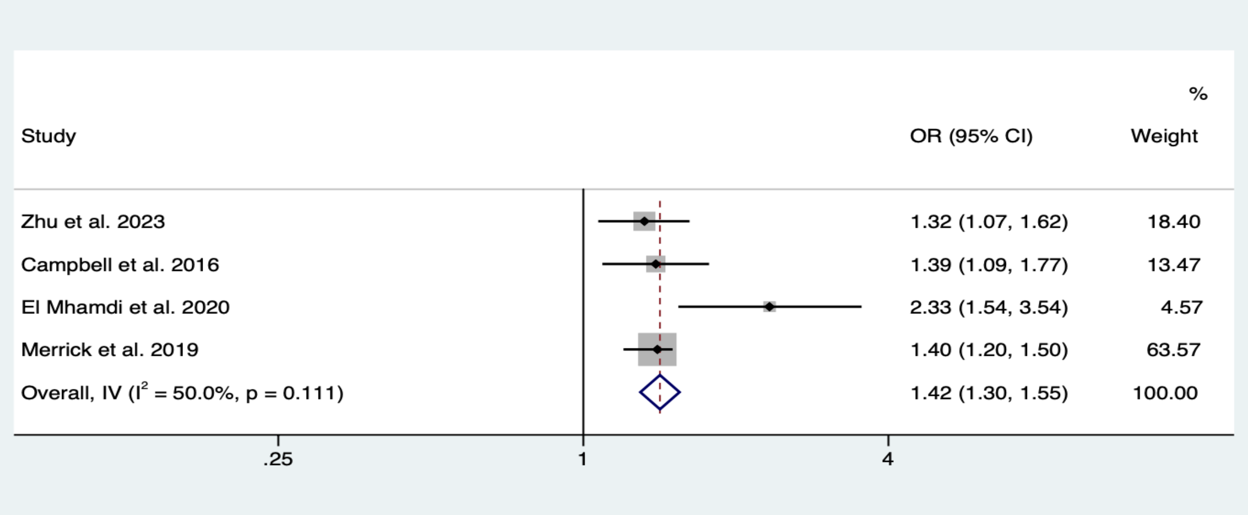

Supplemental Figure 3. Exposure to at least 1 ACE and Outcome of Hypertension

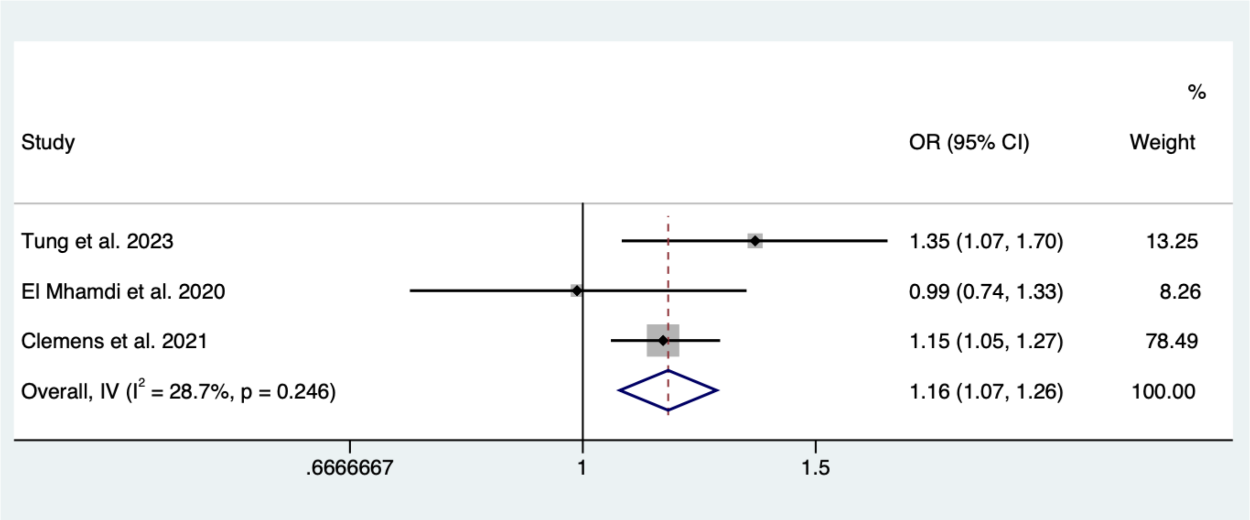

Supplement: Supplementary file 1 — Figure S1: Exposure to at least 1 ACE and Diabetes. Figure S2: Exposure to 3 or more ACEs and Diabetes. Figure S3: One ACE and Hypertension. [file OBR-27-e70095-s001.pdf]
